# Artificial Intelligence in Assisted Reproductive Technology: A New Era in Fertility Treatment

**DOI:** 10.7759/cureus.81568

**Published:** 2025-04-01

**Authors:** Eirini Orovou, Katerina D Tzimourta, Maria Tzitiridou-Chatzopoulou, Antigoni Kakatosi, Antigoni Sarantaki

**Affiliations:** 1 Department of Midwifery, University of Western Macedonia, Ptolemaida, GRC; 2 Department of Electrical and Computer Engineering, University of Western Macedonia, Kozani, GRC; 3 Department of Midwifery, University of West Attica, Athens, GRC

**Keywords:** artificial intelligence (ai), assisted reproductive technology (art), decision support system, deep learning, in vitro fertilization (ivf), machine learning (ml), reproductive medicine

## Abstract

Artificial intelligence (AI) is increasingly applied in assisted reproduction, enhancing success rates and enabling personalized fertility care. The theoretical background of this paper seeks to present an extensive analysis of the key concepts, technological developments, and applications of AI in the field of assisted reproduction. Through a review of existing literature, clinical studies, and practical applications, this paper attempts to demonstrate how AI can contribute to increasing the effectiveness of fertility treatments, address ethical and legal issues, and open new avenues for research and clinical practice in the future.

## Introduction and background

Definitions of infertility and treatment

Infertility is defined as the failure to achieve pregnancy after 12 months of regular sexual intercourse without contraception. Estimates suggest that about one in six couples of reproductive age experience some kind of infertility that may be caused by men, women, or unexplained factors [1]. Approximately 85% of infertile couples have an identifiable cause, while 15% of cases remain unexplained. The male infertility factor accounts for 30% of all cases and concerns sperm quality problems. In comparison, the most common causes of female infertility are reduced ovarian reserve (27%), ovulation problems (14%), and tubal factor (10%) [2].

Assisted reproductive technologies (ARTs), including in vitro fertilization (IVF), have evolved significantly since their first successful application in 1978, leading to a wide range of methods tailored to infertility treatment. The IVF method to be used is decided jointly by the practitioner and the couple after they have been informed of the advantages and disadvantages of each method and based on their individual medical history. The main methods of assisted reproduction include the following: 1) IVF in a natural cycle without medication, 2) IVF with mild stimulation, 3) IVF with full stimulation, and 4) embryo transfer.

Introduction of AI in assisted reproduction

**Figure 1:**
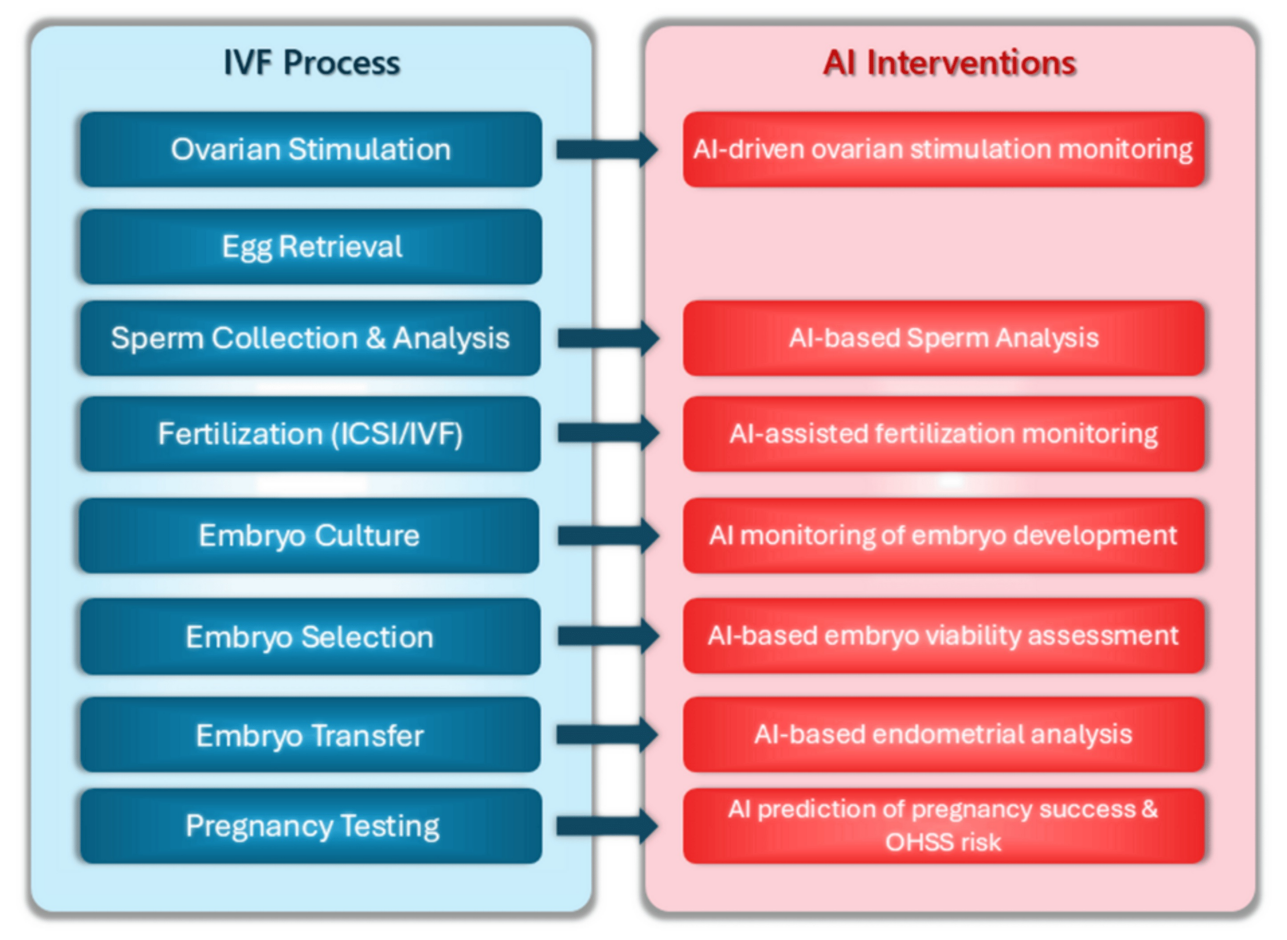
The main steps of the IVF process (blue boxes) and the AI-powered enhancements at specific stages of the IVF process, where AI helps optimize decision-making, improve accuracy, and increase the chances of a successful pregnancy (red boxes) IVF: in vitro fertilization; AI: artificial intelligence; ICSI: intracytoplasmic sperm injection; OHSS: ovarian hyperstimulation syndrome Image credits: This is an original image created by the author Katerina D. Tzimourta

The use of artificial intelligence (AI) in assisted reproduction is a rapidly growing field that is bringing significant improvements in the performance and outcomes of ARTs, such as IVF. The increasing volume of patient data and the diversity of clinical parameters related to fertility are mandating automated analysis by advanced prognostic systems, with the aim of enhancing more informed, accurate, and valid decisions by scientific staff and increasing the success rates of fertility treatments. Key applications of AI in this area include the following: 1) the selection of embryos for evaluation of their quality, prediction of outcomes, e.g., probability of success and overall outcome of different treatments [3], 2) the optimization of treatment protocols through the design of personalized interventions taking into account age, fertility history, and genetic characteristics [4,5], 3) DNA analysis [6], 4) personalized ovarian stimulation to analyze and predict the dose of gonadotropin initiation [7], and 5) assessment of sperm factors such as morphology, motility, DNA integrity, and selection of the best sperm for fertilization [8]. Figure 1 summarizes the key steps of the IVF process (ART) and highlights the points where AI is integrated to enhance efficiency and accuracy.

## Review

Review of AI applications in ART

Algorithms/Systems for Personalized Ovarian Stimulation for IVF

**Figure 2:**
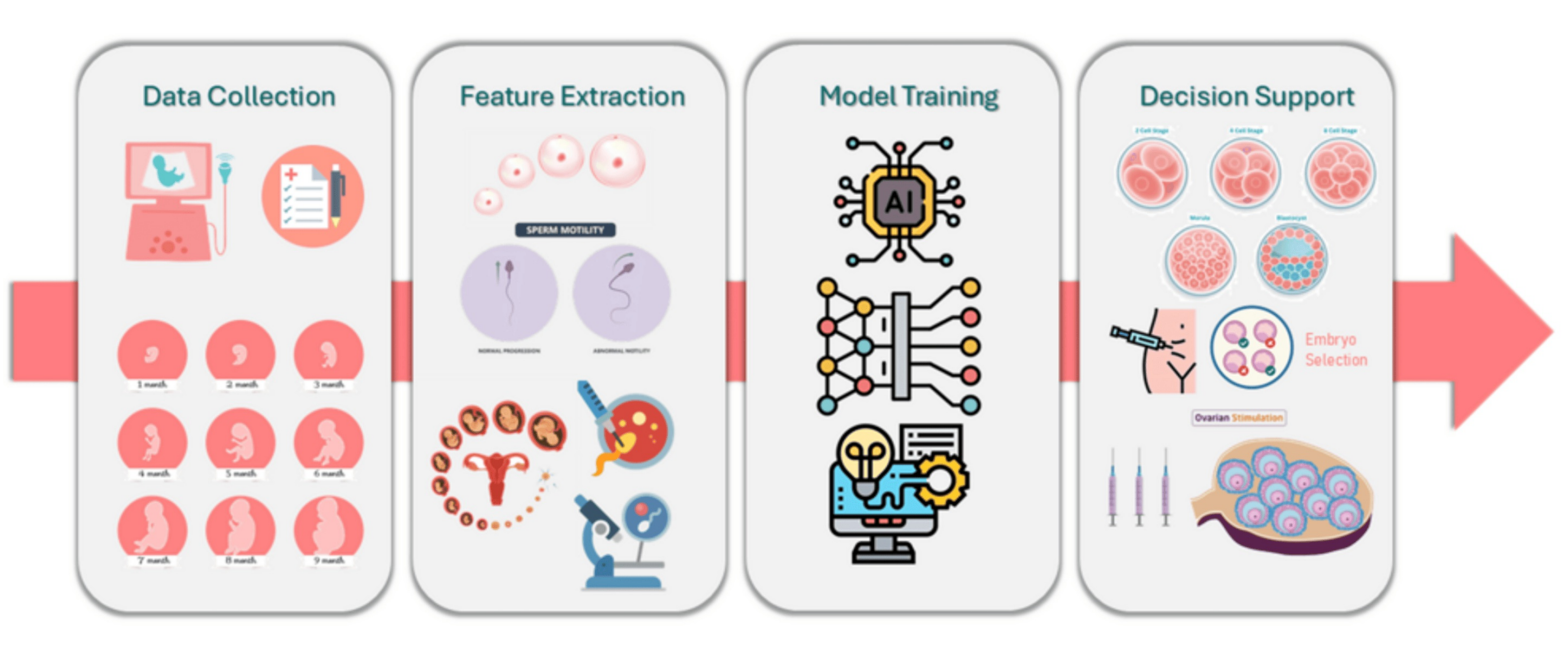
AI workflow in assisted reproduction AI: artificial intelligence Image credits: This is an original image created by the author Katerina D. Tzimourta

Decision support for the daily management of ovarian stimulation: The management of IVF cycles depends on the response of the ovaries to treatment, as assessed in real-time regular check-ups, so that physicians can make decisions based on clinical data and plan the next steps of treatment. Predictive algorithms collect clinical data such as drug doses, estradiol concentrations, follicle ultrasound measurements, and stimulation days as they occur during treatment. They even aggregate data contained in electronic health records of women undergoing IVF and egg cryopreservation to include their demographics, medical history, and infertility evaluation, including diagnosis, laboratory testing for ovarian reserve, and other tests related to the diagnosis of infertility. Hybrid algorithms such as classification and regression trees, random forests (RF), support vector machines (SVMs), logistic regression (LR), and neural networks are recruited to predict critical clinical decisions compared to those based on evidence and made by physicians during ovarian stimulation such as: 1) stopping or continuing stimulation. If the decision was cessation, then the subsequent automated decision was 2) follicle maturation with induction of ovulation or cycle cancellation. If the decision was to continue stimulation, then the subsequent key decisions were 3) determining the recheck and 4) adjusting the dose [9]. Figure 2 depicts the main steps of the integration of AI in ART.

Prediction of the optimal day of activation of final maturation during ovarian stimulation: The goal of ovarian stimulation during IVF is to develop multiple follicles to obtain multiple mature eggs (metaphase II, MII). During ovarian stimulation, doctors are required to make a number of decisions that are critical to the outcome of the cycle, such as the elective protocol, type, and doses of gonadotropins. One of the most important decisions is the timing of the final triggering injection to induce ovarian maturation (triggering). Numerous studies have investigated the relationship between follicle sizes and the effects of MII. Fanton et al. introduced a machine learning model to predict the optimal day for triggering ovulation and estimating mature oocyte yield. The parameters examined were derived from electronic health records, covering 30,278 homologous IVF cycles. These included data such as age, body mass index (BMI), number of previous IVF cycles, antral follicle count (AFC), anti-Müllerian hormone (AMH) levels, baseline estradiol (E2) levels, cycle duration (in days), and daily measurements of follicle sizes and E2 levels recorded during follow-up visits throughout ovarian stimulation. Follicle measurements were classified into six groups based on their diameter: <11-13, 14-15, 16-17, 18-19, and >19 mm. The primary outcome was the number of MII oocytes retrieved, and additional outcomes included two pronuclei and available blastocysts. Models were developed to predict MII outcomes if a patient was activated on the current day (today) compared to the next day (tomorrow). To predict the number of mature MII oocytes retrieved if activation occurred today or tomorrow, linear regression models were used that factored in the number of follicles and E2 levels measured on the day of activation and one day prior to activation, respectively. Finally, an E2 prediction model was developed to predict the next day's E2 levels using the number of follicles and E2 levels measured one day earlier. The combination of these models allowed for a comparison of the MI results based on whether activation was performed today or tomorrow [10].

Automated follicle measurement and follicle maturity prediction: As it has become known, during an assisted reproduction cycle, follicle size correlates with the response of the ovaries to treatment and is an important biomarker for assessing their maturity. However, manual measurement of follicles during ovarian stimulation is time-consuming and subject to high variability between and within the ultrasound operators. Deep learning models such as CR-Unet reduce variability in measuring follicle diameter between clinicians but also support that follicular area is a better biomarker than diameter in assessing follicular maturity. Data for model training consist of the following: 1) 2D ultrasound images of ovaries and follicles measured by diameters and 2) 3D ovarian and follicle images for volume estimation using the virtual organ computer-aided analysis mode of ultrasound, derived from either modified natural cycles or stimulation cycles. In addition, the patient's age, height, weight, and BMI are considered. To achieve image segmentation and ensure maximum accuracy, recurrent neural networks (RNNs) are fitted to the model [11]. The deep learning algorithm and the new biomarker of the follicular region have potential clinical applications in ultrasound monitoring of follicles. Using the network may not increase measurement accuracy for perennially experienced physicians but may provide a reliable and supportive technique for less experienced clinicians. Integrating CR-Unet as ultrasound equipment may speed up the workflow and monitoring of follicles [11].

Determination of gonadotropin starting dose during ovarian stimulation: The selection of an appropriate and personalized dose of gonadotropins at the start of a stimulation cycle is a difficult process as it is linked to the determination of the quality and quantity of oocytes that will be obtained in the laboratory at the end of stimulation and proceed to fertilization. The current approach to determining the dose of gonadotropins is primarily based on the clinician's experience, lacking universal scientific protocols, and on the response to previous attempts. However, this choice poses a risk for women undergoing treatment for the first time. Machine learning prediction models aim to automate dose determination for the initiation of stimulation. After applying the algorithms to evaluate the factors influencing dose selection, he concludes that the four most important factors are age, AMH, AFC, and BMI. Both algorithms have a wide range of applications for modeling classification and regression problems. In particular, the artificial neural network (ANN) model has good modeling performance, in terms of both root mean square error and regression coefficient R-metrics on which their performance is evaluated [12].

Sperm Analysis Systems

Detection and extraction of abnormal morphological characteristics of sperm: Sperm quality is one of the most important parameters for egg fertilization and embryo quality. Semen analysis provides information on the four main characteristics of sperm quality: sperm volume, sperm concentration, sperm motility, and sperm morphology. Deep learning algorithms specialize in detecting and extracting abnormal morphological features of spermatozoa using images of cells as collected by embryology laboratory microscopes, even those with low resolution. As malformations can extend along the entire length of spermatozoa, deep learning models are trained to detect morphological deformities in more than one region of spermatozoa, such as the acrosome, head, and centrioles, in contrast to earlier predictive models that only analyzed individual segments, favoring the acrosome. Convolutional neural networks (CNNs) classify sperm images into two categories: 1) normal and 2) abnormal for different morphological features with high accuracy rates in highly immediate and real-time. Deep learning models rapidly help in selecting the best sperm in the intracytoplasmic sperm injection (ICSI) procedure, as the detection of abnormal tails and necks is usually quite easy for experts. In contrast, the detection of abnormalities in the sperm head is a time-consuming and complicated task. It thus allows an embryologist to quickly decide whether or not to select the analyzed sperm [13]. In ART, it is imperative to identify and select the potentially better sperm that will increase the success rates of ICSI, in turn yielding better quality embryos and resulting in full-term pregnancies and births of healthy children. The development of deep learning models and their adoption by modern assisted reproduction facilities are loosening the hands of embryologists who tend to seek approaches that offer immediate and better solutions to selecting healthy sperm and reducing human error [14].

Evaluation of sperm motility: Sperm motility is intertwined with fertility and requires an automated approach. In the work by Ottl et al. on the automatic assessment of sperm motility, video recordings of mobile sperm from microscopes after preprocessing are fed into machine learning algorithms such as linear support vector regression (SVR), multilayer perceptron (MLP), CNNs, and RNNs, including long short-term memory networks, to extract features for predicting motility. The data used for the experiments come from the VISEM dataset and consist of 85 videos, with each sample annotated for mobility and morphological features. In addition, for each sample, the dataset includes the results of a standard semen analysis and clinical data such as androgen levels, fatty acid levels in spermatozoa or phospholipids, patient age, days of abstinence, and BMI. By comparing the different algorithms for the ensemble mean squared displacement (emsd) feature type, the study shows that the neural network architectures, CNN and RNN, can extract additional information from the features. For the emsd feature type, the SVR is the best, while for content management systems, the MLP algorithm is the best. From the experimental analysis, the progressive motility of spermatozoa is detected most easily, achieving a determination factor of 74%, followed by immobile spermatozoa with a score of 66%. The detection of nonprogressive motility is closer to the baseline value of the mean prediction, achieving a score of only 26% [15].

Estimation of sperm motility velocity: With the contribution of AI methods such as CNN and deep learning, the suitability of a sperm sample for use in any AI process is determined, increasing the success rates of the method. Through static video images obtained from a microscope, an advanced algorithm calculates the speed of head motility, making it easier to select the fastest sperm for fertilization. To train the algorithm, sample-related information such as sperm fatty acids and their composition, ejaculate volume, sperm color, biological fluid dilution time, and patient demographics is also considered. Pretrained CNNs such as Inception, Residual Neural Network, MobileNet, and Visual Geometry Group 16 extract features in image contexts. The novelty is the subsequent application of a faster region CNN neural network to segment the sperm head and identify it from the rest of the image objects. Deep learning neural networks can be used to assess sperm motility consistently and efficiently for the needs of the artificial insemination process, using data including tagged images or videos of sperm and their respective velocities. This approach can be much more automated and accurate than traditional methods of measuring sperm velocity and can improve workflow in clinical practice [16]. Table 1 presents the AI algorithms applied in ART, their advantages, and their limitations.

**Table 1:** AI algorithms integrated into ART along with their advantages and limitations AI: artificial intelligence; CNNs: convolutional neural networks; RF: random forest; SVMs: support vector machines; LR: logistic regression; ANNs: artificial neural networks; LSTM: long short-term memory; ART: assisted reproductive technology; XGBoost: eXtreme Gradient Boosting; LightGBM: Light Gradient Boosting Machine

AI algorithm	Application in ART	Advantages	Limitations
CNNs	Embryo and sperm analysis through imaging	High accuracy in image analysis, automatic feature recognition	Requires large datasets and graphics processing unit training
RF	Treatment outcome prediction and data classification	Good performance in classification and large data analysis	May be prone to overfitting if not properly tuned
SVM	Fertility factor analysis and success prediction	Robustness with small datasets, high generalization	Requires careful selection of input features
LR	Basic tool for pregnancy probability prediction	Simple implementation, easy interpretation of results	Limited accuracy for highly complex data
ANNs	Gonadotropin dose adjustment and treatment optimization	Ability to learn complex relationships between data	High computational power, difficult result interpretation
Gradient boosting (XGBoost, LightGBM)	Implantation outcome and embryo transfer success prediction	Effective on nonlinear data, lower overfitting risk	Needs proper parameter tuning for optimal performance
LSTM	Time-series analysis for treatment progression	Ideal for dynamic data and time-series analysis	High computational cost and sensitivity to data noise

Algorithms/Intrauterine-Based Prediction Systems

Automatic endometrial segmentation and extraction in ultrasound images: The thickness of the endometrium is an essential factor affecting female fertility. In clinical practice, ultrasound imaging is the first choice for investigating uterine and endometrial diseases. However, sometimes, the boundaries of the endometrium are indistinguishable due to poor image resolution and noise in the image. Subsequently, the irregular shape of the endometrium often makes it difficult for doctors to measure its thickness. The maximum thickness of the endometrium can be measured automatically and accurately through automatic segmentation and extraction in ultrasound images. Three-dimensional transvaginal ultrasound images train the 3DU-Net deep learning model to perform endometrial segmentation. The original ultrasound images are subjected to preprocessing such as block matching, 3D filtering, and speckle-reducing anisotropic diffusion. The filtered images combined with the original image create a three-channel image and are now augmented as they enter the 3DU-Net. The midline of the endometrium is extracted with the contribution of the medial axis transform, thanks to which the thickness of the endometrium can be measured automatically [17].

Evaluation of endometrial receptivity to embryo transfer: Endometrial receptivity and good quality embryos contribute to successful implantation. However, the mechanism of implantation in utero remains partly undefined. In recent studies, endometrial shape and overall thickness have been investigated to predict implantation success. In the scientific study by Schuff et al., they focus on using ultrasound imaging and introduce new parameters that measure the outer endometrial layers without manual intervention. EndoClassify, an AI model, is designed to evaluate endometrial characteristics and enhance embryo receptivity. It integrates Attention U-Net for image segmentation and GoogLeNet Inception for image classification. In addition to endometrial images, demographic data such as age, BMI, gravity, and ovarian reserve measurements, and embryological data, including total number of oocytes retrieved, MII, oocyte maturity rate, and fertilization rate, are required. The AI model was trained based on the Asch19 classification using endometrial ultrasound assessment as follows: 1) well-defined ultrasound outer layers, 2) thickness of outer layers, 3) echogenic midline, 4) total endometrial thickness, 5) subechoic intermediate layer positioned between outer layers and midline, and 6) percentage of outer layers relative to total endometrial thickness (equal to or greater than 50% or less than 50%) [18].

Genetic Analysis and Embryo Selection Systems

Evaluation and selection of embryos with low miscarriage potential: In IVF treatments, embryos selected for transfer according to their intended implantation potential, despite having normal morphological and morphomotor profiles, are still at high risk of miscarriage. Thus, the need arises for an accurate and reliable assessment of miscarriage potential, alongside existing technologies that assess implantation potential. The task of evaluating embryos for miscarriage is achieved by machine learning models based on high-quality time-lapse video recordings of embryos at the cell division stage from medically assisted reproduction facilities. The datasets train CNNs that examine the relationships between multiple features in image pixels. CNNs are used to perform automated morphokinetic annotation and implantation prediction [19].

Prediction of embryo euploidy: Couples with serious fertility issues who undergo treatments to resolve them often need further investigation. For this reason, preimplantation testing is recommended. This is the process of detecting structural or gene chromosomal abnormalities in an embryo or genetic disease by examining a few cells, which are taken by biopsy from the blastocyst trophoblast for genetic testing prior to embryo transfer. Preimplantation genetic testing for aneuploidy (PGT-A) has been proposed to be used in women of advanced age, in repeated failed implantation attempts, or in cases of cathartic miscarriages. PGT-A results in the transfer of euploidy (chromosomally normal) embryos, increasing the likelihood of a healthy child being born. It reduces the rates of miscarriage or palindromic pregnancies, the potential for termination in late pregnancy, and significantly reduces invasive methods of prenatal screening and their risks [20]. Nowadays, AI models can predict euploidy embryos that display the normal human chromosomal complement of 46 chromosomes vs. aneuploidy, as the former are associated with improved clinical outcomes. Genetic AI models for predicting the genetic status of embryos are trained using datasets that include thousands of cell mass-focused images obtained through a range of imaging systems, both through microscopy and systemic imaging. AI is offered for image pattern recognition, with the "Embryo Ranking Intelligent Classification Algorithm," which promises to predict with high accuracy and speed the existence of euploidy and the likelihood of embryo implantation. Static images of blastocysts obtained after intracytoplasmic sperm injection (ICSI), along with known outcomes from either preimplantation genetic testing or pregnancy results, were used to train the algorithm. The AI model classifies embryos into two categories: 1) euploid embryos with a good prognosis and 2) aneuploid embryos associated with a poor prognosis and low implantation potential.

Success Prediction Algorithms

Prediction of treatment outcome based on clinical variables: Machine learning algorithms and AI beyond visual images to select higher quality embryos leading to full-term pregnancies have recently been used to predict IVF outcomes based on multiple clinical variables such as ovarian stimulation protocols, women's underlying diseases, medical history, causes of infertility, and patient demographics that are directly or indirectly associated with treatment outcomes. Machine learning models estimate the outcome of treatments by considering clinical data such as patient age and BMI, the number of previous pregnancies, and the number of pregnancies completed in labor. They include data concerning the outcome of ovarian stimulation, such as estradiol (E2) values on days 1 and 6 of ovarian stimulation, duration of ovarian stimulation, total dose of injectable gonadotropins, follicle-stimulating hormone (international units, IU), and luteinizing hormone (IU) used during the cycle. In addition, they take into account parameters related to fertilization, including the number of oocytes obtained at ovulation, the number of mature oocytes (M2), the number of fertilized oocytes, the number of day 3 embryos (D3), and the number of day 3 embryos with the highest quality.

Prediction of pregnancy achievement after fresh embryo transfer: The primary objective of the scientific study by Liu et al. [21] was to highlight the predictive ability of four machine learning models related to pregnancy attainment after fresh embryo transfer. The sample was clinical data from 401 women who underwent frozen embryo transfer.

Predicting the likelihood of pregnancy and multiple pregnancy: It is a fact that AI can handle a large amount of data and is, therefore, suitable for predicting the outcome of IVF through specialized variable analysis models that predict the probability of pregnancy and the probability of multiple pregnancies after transfer of more than one embryo. A recent retrospective study collected 949 medical records of patients who underwent medically assisted reproductive procedures: ovulation and fresh embryo transfer. Six classifiers, including LR, RF, SVM, Light Gradient Boosting Machine, eXtreme Gradient Boosting (XGB), and MLP, were examined on 40 types of data, including patient demographics, IVF cycle, embryo morphological data, and number of transferred embryos. The logic of the prognostic model is very close to the reasoning of clinicians, who first confirm the existence of a pregnancy and then the possibility of a multiple pregnancy after considering the quality and number of transferred embryos. The AI prediction model can provide personalized outcome prediction, right before embryo transfer, and can determine the appropriate number of embryos to be transferred. Furthermore, it can help reduce the risk of multiple pregnancies while still maintaining the optimal probability of pregnancy [22].

Estimating the occurrence of OHSS: Ovarian hyperstimulation syndrome (OHSS) represents a critical condition in ART, characterized by a wide range of clinical manifestations such as ovarian swelling, abdominal distention, nausea, and vascular permeability. OHSS usually occurs after ovarian stimulation with gonadotropins either in the early luteal phase or at the onset of pregnancy when fresh embryo transfer is performed. OHSS can be mild, moderate, or severe, with major implications and may require hospitalization. While clinicians rely on their experience to modify gonadotropin dose to avoid OHSS, machine learning models automated by clinical variables predict incidence among patients and the major factors associated with the syndrome [23].

Diagnosis of endometriosis as a cause of unexplained infertility: Endometriosis is an often painful condition in which endometrial tissue is found in areas outside the uterine cavity, such as the outer part of the uterus, ovaries, fallopian tubes, and peritoneum [24]. ML models such as LR, RF, decision tree (DT), XGB, and hard/softVotingClassifier are techniques for learning a set of clinical parameters directly related to the occurrence of the disease, which are maternal/daughter history of endometriosis, history of surgery for endometriosis, age, BMI, dysmenorrhea, abdominal pain outside menstruation, pain with sciatica, pain during sexual intercourse, back pain outside menstruation, painful defecation, painful urination during menstruation, blood in stool during menstruation, quality of life, and the number of nonhormonal treatments for pain healing [25].

Choice of autologous or heterologous IVF in women of advanced reproductive age: More and more women, especially in developed countries and in recent decades, are choosing to become mothers at an older reproductive age. They often face difficulties in achieving pregnancy as the older they get, the lower the ovarian reserve and the quality of genetic material, so many of them resort to ARTs. For patients and clinicians, the choice between autologous and heterologous IVF is a challenge. Simply put, it is a difficult decision for an older woman to decide to undergo fertility treatments using her own eggs or receive borrowed eggs from an egg donor. Machine learning algorithms such as DTs evaluate the optimal selection criteria for women aged 43-45 with the highest probability of achieving pregnancy with their own eggs or resorting to the option of egg donation. According to the algorithms, the variables associated with the choice of autologous or heterologous IVF are age, BMI, dominant follicles, poor ovarian response to stimulation, fertilized oocytes, number of top-quality embryos, and previous completed pregnancy [26].

Challenges and barriers

Technical Difficulties and Challenges in Integrating AI in Assisted Reproduction

One of the great advantages of AI is its ability to integrate large amounts of heterogeneous data, examine it by identifying patterns, and develop predictive models and selection tools to contribute to decision-making in clinical practice. To achieve this goal, it uses computational analysis of huge datasets, which entails major challenges in the form of data bias, data security, and data access and ownership issues. The main challenges related to data collection and management are as follows: 1) quality and quantity, 2) variety, 3) relevance, and 4) ratio [27]. Table 2 shows a comparison of AI approaches and traditional IVF methods.

**Table 2:** Comparison of AI vs. traditional IVF methods IVF: in vitro fertilization; AI: artificial intelligence

IVF stage	Traditional approach	AI approach	Advantage of AI
Sperm selection	Manual assessment under a microscope	AI-based morphology and motility analysis	Faster, more objective, reduces human error
Ovarian stimulation	Standard hormone-based protocols	AI-driven ovarian stimulation monitoring	Personalized treatment, optimized gonadotropin dosage
Embryo selection	Morphological grading by embryologist	AI-based time-lapse imaging and viability prediction	Higher accuracy in embryo viability prediction
Endometrial receptivity	Ultrasound-based thickness measurement	AI-driven segmentation and receptivity prediction	More precise transfer timing, improved implantation success
Pregnancy prediction	Based on past clinical data and experience	Machine learning models analyzing multivariable patient data	More accurate predictions using large datasets

Development and Training of Machine Learning Models

The application of AI in the field of human reproduction is becoming more and more widespread every day, to contribute directly and effectively to the fertility issues of young couples. However, technical difficulties and challenges for developing and training the predictive models during the experimental process do not cease to exist and need to be addressed immediately before they can be applied in research and clinical practice. The main technical issues concerning the prediction algorithms are as follows: 1) preprocessing and data adequacy, 2) choice of model, 3) validation and testing, 4) retraining, 5) repeatability, 6) staff training, 7) preapplication assessment, and 8) scalability.

Ethical and Legal Considerations in AI-Assisted Reproduction

The use of AI in ART brings up a plethora of ethical and legal issues that must be examined thoroughly. Though AI provides tremendous gains in operational efficiency and predictive power, it also generates issues related to data confidentiality, patient autonomy, algorithm fairness, and legal accountability [28,29]. AI-driven reproductive technologies are based on large volumes of sensitive patient information, such as genetic data, medical history, and fertility-related parameters. Maintaining confidentiality, security, and proper handling of such information is crucial to maintaining patient trust. Compliance with laws such as the General Data Protection Regulation and the Health Insurance Portability and Accountability Act is critical to prevent data breaches and unauthorized disclosure [29]. On the other hand, the use of AI in fertility care demands clear and transparent communication with patients. Patients going through ART need to be made well aware of how AI models assist decision-making, what information is being considered, and the limitations that AI-powered predictions might have. Informed consent must specifically address the use of AI, its accuracy levels, and the risks involved [30].

Moreover, AI algorithms are trained on historical data that may have inherent biases, which result in treatment outcome disparities. When the training dataset is not diverse, AI systems are biased toward specific patient populations, affecting success rates among various groups. Algorithmic fairness and inclusivity must be guaranteed to prevent discrimination and ensure equal access for all patients undergoing fertility treatment [31]. In addition, AI systems also assist in embryo selection, ovarian stimulation protocols, and pregnancy prediction. However, when AI-based recommendations lead to adverse medical outcomes, legal liability becomes an issue. Who is to be blamed: the developers of the software, the fertility clinic, or the physicians relying on AI outputs? Proper legal frameworks are required to address accountability concerns and protect patients and healthcare providers. Therefore, it is necessary to address ethical concerns proactively to ensure that AI positively impacts reproductive medicine rather than weakening it. As AI evolves, a strategy that ensures balance, acknowledging the value of technological advancement while respecting ethical and legal standards, will be critical to the successful and equitable use of AI in fertility treatment [32].

## Conclusions

AI in assisted reproduction offers significant advancements, including improved embryo selection, optimized ovarian stimulation protocols, and enhanced sperm analysis. However, challenges remain in ensuring interpretability, minimizing biases in predictive models, and addressing ethical concerns regarding patient autonomy and data privacy. Despite AI's potential to enhance treatment efficiency and success rates, its integration into clinical practice is hindered by high costs, limited accessibility, and the need for clinician training. Additionally, patient skepticism due to AI’s perceived detachment from personalized care remains a critical barrier. Future research should focus on developing explainable AI models, improving regulatory frameworks, and ensuring equitable access to AI-driven fertility treatments. Addressing these challenges will be crucial in maximizing AI’s benefits while maintaining ethical and patient-centered reproductive healthcare.

## References

[1] (2025). Infertility. https://www.who.int/news-room/fact-sheets/detail/infertility.

[2] Carson SA, Kallen AN (2021). Diagnosis and management of infertility: a review. JAMA.

[3] Choe J, Shanks AL (2023). In Vitro Fertilization. http://www.ncbi.nlm.nih.gov/books/NBK562266/.

[4] Brinsden PR, Brinsden PR (2009). Thirty years of IVF: the legacy of Patrick Steptoe and Robert Edwards. Hum Fertil (Camb).

[5] Kragh MF, Karstoft H (2021). Embryo selection with artificial intelligence: how to evaluate and compare methods?. J Assist Reprod Genet.

[6] Cimadomo D, Chiappetta V, Innocenti F (2023). Towards automation in IVF: pre-clinical validation of a deep learning-based embryo grading system during PGT-A cycles. J Clin Med.

[7] Hariton E, Pavlovic Z, Fanton M, Jiang VS (2023). Applications of artificial intelligence in ovarian stimulation: a tool for improving efficiency and outcomes. Fertil Steril.

[8] Zaninovic N, Rosenwaks Z (2020). Artificial intelligence in human in vitro fertilization and embryology. Fertil Steril.

[9] Letterie G, Mac Donald A (2020). Artificial intelligence in in vitro fertilization: a computer decision support system for day-to-day management of ovarian stimulation during in vitro fertilization. Fertil Steril.

[10] Fanton M, Nutting V, Solano F (2022). An interpretable machine learning model for predicting the optimal day of trigger during ovarian stimulation. Fertil Steril.

[11] Liang X, Fang J, Li H (2020). CR-Unet-based ultrasonic follicle monitoring to reduce diameter variability and generate area automatically as a novel biomarker for follicular maturity. Ultrasound Med Biol.

[12] Hua L, Zhe Y, Jing Y, Fujin S, Jiao C, Liu L (2022). Prediction model of gonadotropin starting dose and its clinical application in controlled ovarian stimulation. BMC Pregnancy Childbirth.

[13] Javadi S, Mirroshandel SA (2019). A novel deep learning method for automatic assessment of human sperm images. Comput Biol Med.

[14] Mashaal AA, Eldosoky MAA, Mahdy LN, Ezzat KA (2023). Classification of human sperms using ResNet-50 deep neural network. Int J Adv Comput Sci Appl.

[15] Ottl S, Amiriparian S, Gerczuk M, Schuller BW (2022). motilitAI: a machine learning framework for automatic prediction of human sperm motility. iScience.

[16] Valiuškaitė V, Raudonis V, Maskeliūnas R, Damaševičius R, Krilavičius T (2020). Deep learning based evaluation of spermatozoid motility for artificial insemination. Sensors (Basel).

[17] (2025). Automatic evaluation of endometrial receptivity in three-dimensional transvaginal ultrasound images based on 3D U-Net segmentation. https://qims.amegroups.org/article/view/95173/html.

[18] Schuff RHA, Suarez J, Laugas N, Ramirez MLZ, Alkon T (2024). Artificial intelligence model utilizing endometrial analysis to contribute as a predictor of assisted reproductive technology success. J IVF Worldwide.

[19] Amitai T, Kan-Tor Y, Or Y (2022). Embryo classification beyond pregnancy: early prediction of first trimester miscarriage using machine learning. J Assist Reprod Genet.

[20] Homer HA (2019). Preimplantation genetic testing for aneuploidy (PGT-A): the biology, the technology and the clinical outcomes. Aust N Z J Obstet Gynaecol.

[21] Liu R, Bai S, Jiang X (2021). Multifactor prediction of embryo transfer outcomes based on a machine learning algorithm. Front Endocrinol.

[22] Wen JY, Liu CF, Chung MT, Tsai YC (2022). Artificial intelligence model to predict pregnancy and multiple pregnancy risk following in vitro fertilization-embryo transfer (IVF-ET). Taiwan J Obstet Gynecol.

[23] Ziaee A, Khosravi H, Sadeghi T, Ahmed I, Mahmoudinia M (2024). Prediction of complicated ovarian hyperstimulation syndrome in assisted reproductive treatment through artificial intelligence. [Preprint]. medRxiv.

[24] Puente E, Alonso L, Laganà AS, Ghezzi F, Casarin J, Carugno J (2020). Chronic endometritis: old problem, novel insights and future challenges. Int J Fertil Steril.

[25] Blass I, Sahar T, Shraibman A, Ofer D, Rappoport N, Linial M (2022). Revisiting the risk factors for endometriosis: a machine learning approach. J Pers Med.

[26] Homanen R, McBride N, Hudson N (2024). Artificial intelligence and assisted reproductive technology: applying a reproductive justice lens. Eur J Women's Stud.

[27] Hirani R, Noruzi K, Khuram H (2024). Artificial intelligence and healthcare: a journey through history, present innovations, and future possibilities. Life (Basel).

[28] Bercovich O, Almog B, Fouks Y (2022). A decision tree analysis applied to women aged 43-45: who should be referred for ovum donation?. Reprod Biomed Online.

[29] Zammit R (2023). Ethical issues of artificial intelligence & assisted reproductive technologies. Int J Prenat Life Sci.

[30] Mapari SA, Shrivastava D, Bedi GN, Pradeep U, Gupta A, Kasat PR, Sachani P (2024). Revolutionizing reproduction: the impact of robotics and artificial intelligence (AI) in assisted reproductive technology: a comprehensive review. Cureus.

[31] Rolfes V, Bittner U, Gerhards H, Krüssel JS, Fehm T, Ranisch R, Fangerau H (2023). Artificial intelligence in reproductive medicine - an ethical perspective. Geburtshilfe Frauenheilkd.

[32] Koplin JJ, Johnston M, Webb AN, Whittaker A, Mills C (2025). Ethics of artificial intelligence in embryo assessment: mapping the terrain. Hum Reprod.

